# Recent advances in the epidemiology and prevention of *Streptococcus pneumoniae* infections

**DOI:** 10.12688/f1000research.22341.1

**Published:** 2020-05-07

**Authors:** Charles Feldman, Ronald Anderson

**Affiliations:** 1 Department of Internal Medicine, Faculty of Health Sciences, University of the Witwatersrand, 7 York Road, Parktown, Johannesburg, 2193, South Africa; 2 Institute of Cellular and Molecular Medicine, Department of Immunology, Faculty of Health Sciences, University of Pretoria, 5 Bophelo Road, Gezina, 0186, South Africa

**Keywords:** herd protection, invasive pneumococcal disease, pneumococcus, pneumococcal conjugate vaccine, prime-boost immunization, pneumococcal polysaccharide vaccine 23, serotype replacement, Streptococcus pneumoniae

## Abstract

The introduction of pneumococcal conjugate vaccines (PCVs) 7 and 13 into national childhood immunization programs in the US in 2000 and 2010, respectively, proved to be remarkably successful in reducing infant mortality due to invasive pneumococcal disease (IPD), resulting in widespread uptake of these vaccines. Secondary herd protection of non-vaccinated adults against IPD has proven to be an additional public health benefit of childhood immunization with PCVs, particularly in the case of the vulnerable elderly who are at increased risk due to immunosenescence and underlying comorbidity. Despite these advances in pneumococcal immunization, the global burden of pneumococcal disease, albeit of unequal geographic distribution, remains high. Reasons for this include restricted access of children living in many developing countries to PCVs, the emergence of infection due to non-vaccine serotypes of the pneumococcus, and non-encapsulated strains of the pathogen. Emerging concerns affecting the elderly include the realization that herd protection conferred by the current generation of PCVs (PCV7, PCV10, and PCV13) has reached a ceiling in many countries at a time of global population aging, compounded by uncertainty surrounding those immunization strategies that induce optimum immunogenicity and protection against IPD in the elderly. All of the aforementioned issues, together with a consideration of pipeline and pending strategies to improve access to, and serotype coverage of, PCVs, are the focus areas of this review.

## Introduction

Worldwide, lower respiratory tract infections (LRTIs) are a major cause of morbidity and mortality. A systematic analysis of the Global Burden of Disease Study 2016 documented that LRTIs, encompassing pneumonia and bronchiolitis, accounted for 2,377,697 deaths (95% uncertainty interval [UI] 2,145,584–2,512,809) overall in 2016, encompassing 652,572 deaths (586,475–720,612) in children younger than 5, and, more strikingly, 1,080,958 (943,749–1,170,638) in adults older than 70 ^1^. Globally, *Streptococcus pneumoniae* (pneumococcus) ** was the leading cause of both morbidity and mortality among the LRTIs, contributing more deaths than all of the other studied etiologies combined ( *Haemophilus influenzae* type b, influenza, and respiratory syncytial virus), and 1,189,937 deaths (95% UI 690,445–1,770,660) were attributed to the pneumococcus. The authors concluded that, although progress had been made in addressing the burden of LRTIs, it has not been equal across all locations and that more effort needed to be focused on the elderly since almost three quarters of LRTI deaths occurred in older children and adults, and the mortality rate is particularly high in those older than 70. Furthermore, the authors indicated that risk factors, including comorbidities, which put the elderly at risk of LRTIs, needed to be explored further. Moreover, the impact of the introduction of the pneumococcal conjugate vaccine (PCV) on the elderly in low- and middle-income countries in particular needed to be evaluated.

This review explores recent data regarding the global burden of *S. pneumoniae*, the changing recommendations for, and effects of, the introduction of PCV, particularly in childhood immunization programs but also in adults, and describes what is new on the horizon for future pneumococcal vaccination.

## Burden of pneumococcal disease

There appears to be a changing burden of pneumococcal infection, at least in certain parts of the world, and such is the decline in the rate of pneumococcal infections that, in the US, this microorganism is now believed to account for less than 10 to 15% of cases of community-acquired pneumonia (CAP) ^2^. For example, in a 2015 active population-based surveillance of hospitalized CAP in adult patients in the US (EPIC Study), the most common pathogens were rhinovirus (9% of patients), influenza virus (6%) and the pneumococcus, which constituted 5% of the documented cases ^3^. In Europe, on the other hand, the proportion of cases due to the pneumococcus is higher than that reported for the US and has been attributed to differences in vaccine practices and the habit of smoking ^2^. For example, in a recent structured literature review that documented a varying but high burden of pneumococcal CAP in different countries in Europe, the burden occurred particularly among the elderly with comorbidities, despite the use of the 23-valent pneumococcal polysaccharide vaccine (PPV23) ^4^. The importance of comorbidities in the burden of pneumococcal infections has also been documented in the US, where rates of pneumococcal disease in at-risk and high-risk adults are much higher than in healthy adults ^5^.

One of the reasons that discrepancies exist in the frequencies of the documented etiologies in CAP in the different studies may relate to the definition of the etiology ^6^. Blood culture is often considered the “gold standard” of microbiological diagnosis in bacterial CAP but lacks sensitivity, and although commercially available urine antigen detection tests for the pneumococcus and *Legionella pneumophila* have better efficacy, they still have a sensitivity of only about 70 to 80% ^6^. Recent advances have been made in the molecular methods for the detection of putative pathogens; however, it has been said that these novel diagnostic methods are challenging in the absence of a true gold standard and that studies using strict case definitions, radiographic review, and application of these tests to control specimens may help elucidate their validity ^6^.

A recent study that evaluated the prevalence of pneumococcal pneumonia in patients hospitalized with CAP in Thailand used quantitative polymerase chain reaction of upper respiratory tract secretions and a commercially available urine antigen test (UAT) (BinaxNOW) together with Bayesian latent class modelling and conventional (BLCM) analysis ^7^. The BLCM analysis suggested a greater than 25% higher prevalence of pneumococcal pneumonia than that estimated by a conventional approach when assuming UAT to be the gold-standard reference test. In the EPIC Study, most of the pneumococcal infections were documented by using the BinaxNOW ^3^. However, when a novel serotype-specific urine antigen detection assay was applied to residual stored urine specimens from this study, it substantially increased the documentation of pneumococcal infection among the CAP cases, even though it diagnosed only serotypes contained in PCV13 ^8^. Another prospective observational surveillance study of hospitalized CAP cases across 10 geographically dispersed cities in the US used cultures of the respiratory tract and normally sterile sites as well as the same serotype-specific urine antigen detection assay, and the authors concluded that, despite the uptake of PCV programs in children and associated herd protection in adults, there remained a persistent burden of PCV13-type CAP in the US ^9^.

Other studies and reviews published in the last four years from different parts of the world, including Australia ^10, 11^ and the Asia Pacific region ^12^, Canada ^13, 14^, Latin America ^15– 17^, and the Caribbean ^17^, have documented similar findings. Namely, there is an ongoing high rate of pneumococcal pneumonia in all of these regions; in more recent years, this is often the case despite decreased rates associated with herd protection in adults from implementation of childhood PCV immunization as well as a residual burden of vaccine-serotype pneumonia and invasive pneumococcal disease (IPD), as described above.

## Pneumococcal vaccination

A number of recent reviews of pneumococcal vaccination have described the differences between PPV23, the 23-valent polysaccharide vaccine, and PCV13, the 13-valent pneumococcal polysaccharide conjugate vaccine, the two vaccines that are registered for use in adults. These reviews recognize (i) the ability of the conjugate vaccine to inhibit carriage of vaccine serotypes in vaccinated children, resulting in not only direct protection but also indirect herd protection among unvaccinated children and adults ^18^, (ii) the importance of nasopharyngeal carriage and an understanding of the immunological mechanisms that confer protection against colonization and facilitate novel vaccine development and evaluation ^19^, and (iii) the need to study memory B-cell responses more intensively to fully understand long-term protection with PCV ^20^.

### Herd protection

Introduction of the various conjugate vaccines into routine childhood vaccination programs, including PCV7 ^21, 22^, PCV10 ^23, 24^, and PCV13 ^25– 27^, was shown to result in a decrease in serotype-specific IPD or pneumococcal pneumonia or both in the vaccinated individuals and adults, particularly the elderly, the latter via indirect herd protection. These include studies of PCV7 ^22^ and PCV10 ^24^ in low- and middle-income countries. Clearly, however, the results of additional studies from developing countries, several of which are ongoing, are necessary to accurately determine whether the impact of introduction of PCV is comparable to that in the developed world ^28^. The indirect effects of PCV on IPD or pneumococcal pneumonia (or both) in older adults appear to be dependent on PCV coverage rates, time from PCV implementation and differed according to the specific PCV used ^29, 30^.

### Serotype replacement disease

It is clear from a myriad of recent single-country studies (Ireland ^31^, England and Wales ^32^, Japan ^33^, Canada ^34, 35^, and Portugal ^36^) and from surveys and systematic reviews/meta-analyses ^37, 38^ that, with the progressive introduction of PCV in childhood immunization programs and the associated direct and indirect benefits of reducing vaccine serotype disease, serotype replacement disease/infection has also occurred in both children and adults, in some cases mainly in adults and especially in older adults. This has resulted from an increase in serotypes that are not covered by the specific vaccine introduced (for example, increase in unique PCV13 serotypes with introduction of PCV7 or increase in unique PPV23 serotypes, and non-vaccine serotypes [NVTs], after introduction of PCV13). Clearly, there are geographical differences in serotype replacement and there is a striking difference between the UK and the US ^39^. In the UK, as in Europe, NVT IPD has occurred mainly in older adults, whereas in the US NVT IPD has remained relatively stable in young children and older people. The authors contend that it is difficult to fully understand the reason (or reasons) for these differences since the circumstances in the two countries are otherwise very similar.

In some countries, such as Canada ^35^ and Portugal ^36^ and others ^40^, there has also been an increase in serotype 3 pneumococcal infection despite the introduction of PCV13, which contains serotype 3, and there is a lower efficacy of PCV13 in combatting serotype 3 as compared with other serotypes ^40^. These factors have led some of these authors to suggest that changes in vaccination strategies in adults need to be considered in order to build on the successes of PCVs in children, which are potentially being undermined by serotype replacement ^31, 34, 37^.

## Recommendations for vaccination in adults, including the elderly

Currently, two vaccines are registered for use in adults, namely PPV23 and PCV13 ^41, 42^. Recommendations have been made for their use on the basis of age and risk factors in adults, although changes in the exact recommendations and the dosing intervals have been made over the years, as reviewed elsewhere ^41– 43^. However, new changes in the recommendations were recently published by the Centers for Disease Control and Prevention (CDC) (US) ^44^. This relates primarily to the use of both PCV13 and PPV23 in adults who are at least 65 years old.

The earlier recommendation by the CDC for the dual use of these two vaccines in adults who are at least 65 years old ^45^ was made in 2014 in recognition of the following: (i) despite herd protection in adults, there remained a residual burden of PCV13 serotype pneumococcal infection in adults; (ii) an increase in unique PPV23 serotype infection due to serotype replacement disease in adults; (iii) the importance of the burden of non-bacteremic pneumococcal pneumonia and new evidence from the Community-Acquired Pneumonia Immunization Trial in Adults (CAPiTA) that PCV13 was effective against non-bacteremic infections ^46^; (iv) data that suggested that although PPV23 was efficacious in preventing IPD among healthy young adults and generally healthy older individuals, studies of its efficacy in non-bacteremic infections were contradictory; and (v) acquisition of immunogenicity data supporting the use of PCV13 followed by PPV23 data (“prime boost” phenomenon), even in immunocompromised individuals ^46– 49^.

In the case of pneumococcal immunization, the recommended “prime boost” immunization strategy involves initial immunization with a single dose of PCV13 followed weeks or months later by a single dose of PPV23 in vaccine-naïve individuals. The perceived advantages of this strategy, investigated originally in vaccine-naïve elderly persons ^48^, are cost-effectiveness, maximizing the number of vaccine serotypes and augmentation of immunogenicity. Sadlier *et al.* have also demonstrated the efficacy of the PCV13/PPV23 “prime boost” immunization strategy in HIV-infected persons ^47^. In that study, HIV-infected persons (n = 64) were stratified to receive PCV13 followed 4 weeks later by PPV23 (n = 31 persons) or PPV23 at week 4 only (n = 33 persons). Measurement of serotype-specific IgG antibodies and functional opsonophagocytic antibodies was performed 8 and 28 weeks after administration of PPV23 ^47^. The authors reported that the “prime boost” group was more likely to achieve increases of between two- and four-fold in the serum concentrations of serotype-specific and opsonophagocytic antibodies, respectively, at both time intervals tested ^47^.

Despite the apparent potential of the pneumococcal “prime boost” immunization strategy, several important concerns remain. These include (i) identifying the optimum interval between administration of PCV13 and PPV23, (ii) the duration of immunological protection, (iii) induction of immune hyporesponsiveness due to subsequent “boosting” with PPV23, (iv) failure of this strategy to induce persistent augmentation of protective antibody responses in clinical settings other than HIV infection ^50^, and (v) absence of compelling clinical trial data demonstrating efficacy in the prevention of severe pneumococcal infection.

Two opinion pieces were subsequently published in *JAMA Internal Medicine* by experts debating these recommendations, either supporting these recommendations or citing a potential lack of evidence for this recommendation, the complexity of the recommendations, and associated increased costs ^51, 52^. Nevertheless, it was recognized at that time that these recommendations would need to be re-looked at in the future as herd protection increased. This was done in 2019, when the recommendations of the CDC were again changed; the indication was that all adults at least 65 years of age should continue to receive PPV23 but that there should be shared decision making regarding preceding use of PCV13 in this age group among patients who do not have an immune-compromising condition, cerebrospinal fluid leak, cochlear implant, or increased risk of exposure to PCV 13 serotypes ^44^.

## Ongoing controversies in adult pneumococcal vaccination

Nevertheless, there is still considerable debate about the best way to protect adults, including the elderly, against pneumococcal infection, and many questions need to be answered. There is also recognition that there are differences in different geographical regions with regard to both the residual burden of PCV-serotype disease following childhood vaccination and the extent of serotype replacement disease in both adults and children ^53^. As indicated previously, it is not completely clear why Europe and the US differ in both of these regards ^53, 54^. Furthermore, there needs to be a consideration of whether the herd protection of adults through childhood vaccination is as good a way of protecting adults as actively immunizing them ^54, 55^. It has even been suggested that some countries appear to have reached the limits of their herd protection ^56^, and a study from Switzerland even showed an increase in all-cause pneumonia and pneumococcal pneumonia in adults following introduction of pneumococcal vaccination in children ^57^.

There are additional issues that need to be addressed in the overall consideration of both PCV13 and PPV23 vaccination in adults. In this context, the ongoing incidence of PCV-serotype infections, particularly non-bacteremic infections, may be underestimated. For example, one study from Spain in immunocompetent adults hospitalized with CAP noted that infections with serotypes included in PCV13 still occurred commonly in both healthy individuals and those with underlying conditions, following introduction of PCV13, when this was diagnosed by using the serotype-specific UAT ^58^. Another study from Spain confirmed the value of the commercially available UAT to increase the detection rate of pneumococcal infections ^59^. In addition, while some have questioned the use of PPV23 in adults, largely because of possible hyporesponsiveness occurring after its use ^60^, this needs to be counterbalanced by recent studies indicating a potential benefit of PPV23 against both IPD and non-bacteremic pneumococcal infections ^61– 63^. Clearly, many factors influence the incidence of pneumococcal disease and the response to pneumococcal vaccination, including personal conditions, geographical and ethnic factors, and social risks, not all of which have been considered ^64^. Certainly, many countries have an aging population, and adults, both young and older with underlying medical conditions, have an increased risk of pneumococcal pneumonia and IPD ^57, 65, 66^, and although declines in pneumococcal disease have occurred in older adults and in those with comorbidities following the introduction of childhood PCV13 immunization, these declines have been attenuated ^66^. So the holy grail is, what is the future of adult pneumococcal vaccination ^67^?

## New-generation pneumococcal conjugate vaccines

Important concerns driving the development of new-generation PCVs include the following:

- An increasing realization (as covered in detail above) that the extent of herd protection provided by existing vaccine coverage (PCV7/PCV13) appears to have plateaued in many geographic regions ^56^. This limitation of existing vaccines necessitates the development of novel extended-coverage PCVs to counter the threat posed by NVTs and also to potentiate the immunogenicity of the PCV13 serotypes 4, 19F, and 19A and serotype 3 in particular ^56, 68^. Although these novel vaccines will contribute to the efficacy of pneumococcal immunization programs in children, they are expected to offer considerable benefit to the elderly and other groups at high risk for the development of pneumonia and IPD. PCV15 and PCV20 are new-generation vaccines that fall into this category; the major beneficiaries of advances in pneumococcal immunization strategies have been children living in high-income countries where PCV7, PCV10, and PCV13 have been enthusiastically and effectively included in national childhood immunization programs. In many instances, children living in low-income countries have experienced similar benefit, largely through the efforts of the “Global Alliance for Vaccines and Immunization”, now known as “Gavi, the Vaccine Alliance”. This is an international philanthropic organization that, through negotiation and sponsorship, has enabled access to vaccines, including PCVs, for children living in some of the world’s poorest countries. However, in spite of these efforts, it has been estimated that 500,000 pneumococcal deaths occurred in children between 1 and 59 months of age in 2015 ^69, 70^. Most of these deaths occurred in developing countries, and India, Nigeria, the Democratic Republic of Congo, and Pakistan accounted for 50% of these ^69, 70^. Mercifully, a more affordable new PCV10 targeting serotypes (1, 5 , 6A, 6B, 7F, 9V, 14, 19A, 19F, and 23F) of the pathogen, which are common in African and Asian countries, has been granted prequalification status (“facilitated access to medicines that meet the unified standards of quality, safety and efficacy”) by the World Health Organization ^71^. The vaccine, known as Pneumosil ^®^, will be available to low- and middle-income countries.

### PCV20

Of the two third-generation extended-coverage PCVs (PCV15 and PCV20), the latter is in the most advanced stages of clinical evaluation (safety and immunogenicity). The vaccine was designed primarily for immunization of adults. In addition to the capsular polysaccharides present in PCV13, which are derived from serotypes 1, 3, 4, 5, 6A, 6B, 7F, 9V, 14, 18C, 19A, 19F, and 23F, the seven new serotypes contained in PCV20 are 8, 10A, 11A, 12F, 15 B/C, 22F, and 33F ^72^. Inclusion of the new serotypes confers additional protection against fatal disease (8, 10A, 11A, 15B/C, 22F, and 33F), infection with antibiotic-resistant strains of the pathogen (11A, 15B/C, 22F, and 33F), and meningitis (10A, 15B/C, 22F, and 33F) ^72^. PCV20 has successfully completed phase 1 and phase 2 clinical evaluations. The phase 1 trial (randomized, controlled observer-blind, two-arm parallel design) assessed the safety, tolerability, and immunogenicity in healthy, immunocompetent adult humans (n = 66) (age of 18 to 49 years) with no history of pneumococcal immunization ^73^. Participants were randomly assigned to receive a single intramuscular dose of PCV20 alone (n = 33) or a licensed combination of tetanus, diphtheria, cellular pertussis vaccine. Immunogenicity (opsonophagocytic antibody titers and concentrations of capsular-specific IgG antibodies) was measured 4 weeks post-immunization and the overall safety profile over 6 months ^73^. Impressive immunogenicity and safety were evident on completion of this trial.

The phase 2 trial included 444 vaccine-naïve, immunocompetent adults (age of 60 to 64 years) randomly assigned to receive either PCV13 or PCV20 (single intramuscular injection) followed by assessment of immunogenicity and safety ^74^. No safety concerns were evident, while immunogenicity was impressive and significant increases in opsonophagocytic antibody titers were detected for all capsular polysaccharides present in PCV20. Geometric mean fold increases from baseline of 6.1 to 68.6 and 9.0 to 112.2 were detected for antibodies targeting capsular polysaccharides present in PCV 13 only and for the additional capsular polysaccharides in PCV20, respectively ^74^. On the basis of these findings, the US Food and Drug Administration awarded “breakthrough therapy status” to PCV20 (expedited evaluation and development).

Subsequently, the Pfizer Vaccine R&D Program has initiated three phase 3 clinical trials, all scheduled for completion in early 2020. Notwithstanding safety issues, the primary phase 3 trial (ClinicalTrials.gov Identifier: NCT03760146) ^72^, to which 3880 healthy, vaccine-naïve adults have been recruited, is focused on (i) a comparison of the immune responses to PCV20 and dual PCV13/PPV23 immunization in subjects at least 60 years old and (ii) the immunogenicity of PCV20 in persons 18 to 59 years old ^72^.

The second phase 3 trial (ClinicalTrials.gov Identifier: NCT03835975) ^72^ is focused on the evaluation of safety and immunogenicity of PCV20 in elderly adults (n = 785, at least 65 years old) who had received prior immunization with PCV13 alone (≥6 months earlier) or with PPV23 alone (≥1 year to <5 years earlier) or PCV13 followed by PPV23 (≥1 year earlier) ^72^.

In addition to providing additional safety data, the third phase 3 trial (ClinicalTrials.gov Identifier: NCT03828617) ^72^, to which 1610 adults (age of 18 to 49 years) with no history of pneumococcal immunization have been enrolled, is designed to evaluate the immunogenicity of three different lots of PCV20.

Considering estimates that about 79,000 of the 1.1 million cases of adults hospitalized with CAP in the US annually are caused by serotypes of the pneumococcus represented in PCV20, Thompson *et al.* recently proposed that a significant number of these cases may be prevented by immunization with this vaccine ^73^.

### PCV15

The second third-generation PCV, PCV15, produced by Merck Vaccines, consists of the 13 pneumococcal serotypes present in PCV13 (1, 3, 4, 5, 6A, 6B, 7F, 9V, 14, 18C, 19A, 19F, and 23F) together with the additional serotypes 22F and 33F ^75^, which (as mentioned above) are associated with infection caused by antibiotic-resistant strains of the pneumococcus as well as with severe disease, including meningitis. Unlike PCV20, which appears to target the prevention of severe pneumococcal disease in adults, PCV15 will be used in both the pediatric and adult settings. The safety and efficacy of PCV15 have been demonstrated in several phase 2 clinical trials undertaken in children ^75^ and adults ^76, 77^ and in a pre-clinical experimental animal study ^78^.

In a pediatric setting, Greenberg *et al.* recently reported the results of a multicenter (n = 58), phase 2 clinical trial (ClinicalTrials.gov Identifier: NCT01215188) encompassing five countries (US, Canada, Finland, Israel, and Spain) and 1148 healthy infants ^75^. Relative to the comparator vaccine (PCV13), PCV15 had an acceptable safety profile, and immunogenicity (four doses of vaccine administered at 2, 4, 6, 8, and 12 to 15 months of age) was also comparable to that of PCV13 ^75^.

Two phase 2 trials undertaken by the same group investigated the safety and immunogenicity of PCV15 relative to those of PCV13 in adults ^76^. The first of these (ClinicalTrials.gov Identifier: NCT02547649), to which healthy adults (n = 690) younger than 50 years were enrolled, compared the safety and immunogenicity of PCV15 with those of PCV13, which were found to be comparable ^76^. The second study, to which adults (age of at least 65 years) who had previously been immunized with PPV23 were enrolled (n = 253), compared the safety and immunogenicity of PCV15 with those of PCV13 ^77^. The authors reported that PCV15 was “generally well tolerated and induced high levels of IgG and opsonophagocytic antibodies to all 15 serotypes included in the vaccine when given as a single dose to adults at least 65 years of age previously vaccinated with PPV23” ^77^.

The authors of the aforementioned studies contend that PCV15 will provide necessary additional protection against the threat of IPD posed by serotypes 22F and 33F, which has emerged following the introduction of PCV13.

## Serotype-independent pneumococcal vaccines

Despite the remarkable public health impact of PCVs in infant immunization, ongoing challenges persist, specifically those presented by serotype replacement with NVTs of the pneumococcus as well as the emergence of non-encapsulated strains of the pathogen. In the opinion of many experts, the development of vaccines based on highly conserved and immunogenic protein antigens remains the “holy grail” of universal pneumococcal immunization ^79^. Potential immunization strategies of this type include (i) vaccines based on single recombinant pneumococcal proteins or combinations of these, (ii) combining recombinant protein antigens with PCVs, and (iii) attenuated, non-encapsulated whole-cell vaccines, in which immunogenic surface proteins of the pathogen are exposed ^80^. An example of the latter type of vaccine is that derived from a non-proliferating and non-encapsulated strain of the pneumococcus (RM200 RXIE Pdt ΔlytA) in which the *lytA* (autolysin) and *ply* genes are deleted and inactivated, respectively ^81^.

However, to the best of our knowledge, no vaccine of any of the aforementioned types has progressed beyond the early stages of clinical evaluation. Two PCV/recombinant protein vaccines have reached phase 2 evaluation. These are (i) a vaccine based on PCV13 in combination with two recombinant pneumococcal proteins (pneumolysin toxoid and pneumococcal histidine triad protein D ^82^) and (ii) a variant of PCV10, known as PhiD-CV (which consists of serotypes 1, 4, 5, 6B, 7F, 9V, 14, 18C, 19F, and 23F with eight capsular polysaccharides conjugated to protein D of non-typeable *H. influenzae* and the others to tetanus or diphtheria toxoid), in combination with the same two pneumococcal proteins ^83^.

## Conclusions

The ongoing development and uptake of PCVs into national childhood immunization programs in many countries have impacted enormously on the public health threat posed by severe pneumococcal disease. Nevertheless, the burden of pneumococcal disease remains high in many countries, including those with compulsory childhood access to PCVs. Although a major contributing factor remains the limited serotype coverage provided by PCVs and the associated emergence of IPD caused by NVTs of the pneumococcus, other factors are also at play. These include recognition of disease caused by non-encapsulated strains of the pathogen, reaching the ceiling of herd protection, and the uncertainty surrounding the efficacy of current immunization practices in high-risk groups, compounded by global population aging and associated immunosenescence and comorbidity. Although expanded serotype coverage vaccines have been targeted for expedited approval, issues such as affordability and vaccine efficacy in the face of ongoing serotype replacement are likely to remain significant concerns. The search continues for a serotype-independent vaccine to counter the global burden of pneumococcal disease.
